# Studying the effect of chloroquine on sporozoite-induced protection and immune responses in *Plasmodium berghei* malaria

**DOI:** 10.1186/s12936-015-0626-2

**Published:** 2015-03-26

**Authors:** Else M Bijker, Krystelle Nganou-Makamdop, Geert-Jan van Gemert, Fidel Zavala, Ian Cockburn, Robert W Sauerwein

**Affiliations:** Department of Medical Microbiology, Radboud University Medical Center, P.O. Box 9101, 6500 HB Nijmegen, The Netherlands; Johns Hopkins Malaria Research Institute and Department of Molecular Microbiology and Immunology, John Hopkins Bloomberg School of Public Health, Johns Hopkins University, Baltimore, MD 21205 USA; Current address: Vaccine Research Centre; National Institutes of Health, 40 Convent drive, Bethesda, MD 20892 USA; Current address: John Curtin School of Medical Research, Australian National University, GPO Box 334, Canberra City, ACT 2600 Australia

**Keywords:** Chloroquine, Sporozoite immunization, *P. berghei*, T cells, Immunity, Protection

## Abstract

**Background:**

Sporozoite immunization of animals and humans under a chemo-prophylactic cover of chloroquine (CPS-CQ) efficiently induces sterile protection against malaria. In humans, CPS-CQ is strikingly more efficient than immunization with radiation attenuated sporozoites (RAS), raising the hypothesis that this might be partially due to CQ. Chloroquine, an established anti-malarial drug, is also well known for its immune modulating properties including improvement of cross-presentation. The aim of this study was to investigate whether co-administration of CQ during sporozoite immunization improves cellular responses and protective efficacy in *Plasmodium berghei* models.

**Methods:**

A number of experiments in selected complimentary *P. berghei* murine models in Balb/cByJ and C57BL/6j mice was performed. First, the effect of CQ administration on the induction of protection and immune responses by RAS immunization was studied. Next, the effect of CQ on the induction of circumsporozoite (CS) protein-specific CD8^+^ T cells by immunization with *P. berghei* parasites expressing a mutant CS protein was investigated. Finally, a direct comparison of CPS-CQ to CPS with mefloquine (MQ), an anti-malarial with little known immune modulating effects, was performed.

**Results:**

When CQ was co-administered during immunization with graded numbers of RAS, this did not lead to an increase in frequencies of total memory CD8^+^ T cells or CS protein-specific CD8^+^ T cells. Also parasite-specific cytokine production and protection remained unaltered. Replacement of CQ by MQ for CPS immunization resulted in significantly reduced percentages of IFNγ producing memory T cells in the liver (p = 0.01), but similar protection.

**Conclusions:**

This study does not provide evidence for a direct beneficial effect of CQ on the induction of sporozoite-induced immune responses and protection in *P. berghei* malaria models. Alternatively, the higher efficiency of CPS compared to RAS might be explained by an indirect effect of CQ through limiting blood-stage exposure after immunization or to increased antigen exposure and, therefore, improved breadth of the immune response.

**Electronic supplementary material:**

The online version of this article (doi:10.1186/s12936-015-0626-2) contains supplementary material, which is available to authorized users.

## Background

Whole sporozoite immunization approaches, such as chloroquine chemoprophylaxis and sporozoites (CPS-CQ) and radiation-attenuated sporozoites (RAS), efficiently induce protection in murine malaria models [1-3]. In humans CPS-CQ is about 20 times more efficient than RAS, requiring bites from a total of 45 *versus* 1,000 mosquitoes, respectively [4-6]. Moreover, long-lasting immune responses after CPS-CQ immunization in studies with mice [7] and healthy human volunteers [8] go together with protracted protection. Several murine studies have demonstrated the essential role of CD8^+^ T cells in sporozoite-induced pre-erythrocytic immunity [9-14,2]. Generation of these CD8^+^ T cells against pre-erythrocytic antigens requires cross-priming by dendritic cells [15].

CQ has since its discovery in 1934 been used widely and successfully as anti-malarial, until resistance developed [16], and has more recently been explored for treating cancer and viral infections [17,18]. Interestingly, CQ was also shown to enhance cross-presentation of soluble antigens and non-replicating influenza virus *in vitro* [19,20]. Moreover, *in vivo* cross-priming of naïve CD8^+^ T cells with soluble ovalbumin was more effective in CQ–treated compared to untreated mice [21], and CQ improved the induction of influenza-specific cytolytic T cells in mice [20]. In humans, co-administration of CQ with a hepatitis B vaccine booster significantly increased the number of virus-specific IFNγ-producing CD8^+^ T cells [19].

This study was based on the hypothesis that CQ, which affects endosomal acidification and the degradation and transport of antigens to the cytosol [22,23], could favour cross-presentation of pre-erythrocytic *Plasmodium* antigens and thereby contribute to the efficient induction of immune responses and protection by CPS-CQ immunization. The first topic of investigation was the effect of CQ on immunization by RAS, an established immunization model relying on CD8^+^ T cell responses, thus potentially benefiting from improved cross-presentation. CD8^+^ T cells recognizing the immunodominant circumsporozoite (CS) protein can mediate protective immunity [24]. Therefore, the effect of CQ on the induction of CS-specific CD8^+^ T cells by immunization with *P. berghei* parasites that express a mutant CS protein containing the model SIINFEKL H-2K^b^ epitope was investigated next. Finally, a direct comparison was performed betweeen CPS-CQ and CPS with mefloquine (MQ), an anti-malarial with little known immune modulating effects [25-27], not including improvement of cross-presentation. Akin to CQ, MQ induces arrest of early blood-stage parasites without an effect on pre-erythrocytic parasite stages, allowing full liver-stage development and brief exposure to early blood stages. By performing experiments in these selected complimentary *P. berghei* murine models, the aim of this study was to explore the effect of CQ on protection and T cell responses after whole sporozoite immunization.

## Methods

### Mice and parasites

Balb/cByJ and C57BL/6j mice (6 to 8 weeks old) were purchased from Elevage-Janvier (Le Genest Saint Isle, France). These mouse strains were selected based on extensive experience with these strains for malaria immunization studies [28]. The following parasites were used: *P. berghei* (ANKA strain) wild type parasites and *P. berghei* CS5M parasites in which the endogenous CS gene had been replaced with a modified circumsporozoite gene expressing the H-2Kb restricted SIINFEKL [15]. Sporozoites were obtained by dissection of the salivary glands of infected female *Anopheles stephensi* mosquitoes 21–29 days after a blood meal on infected mice. All animal studies and procedures performed in the Netherlands were approved by the Ethical Committee on Animal Research of the Radboud University Nijmegen (RU-DEC 2009–179, 2009–225, 2010–115, 2010–135). Mice were housed at the Central Animal Facility in Nijmegen and received a standard diet and water *ad libitum*. All animal procedures in the United States of America were approved by the Institutional Animal Care and Use Committee of the Johns Hopkins University (Protocol Number MO10H167) and followed the National Institutes of Health guidelines for animal housing and care.

### Immunization schedules, sporozoite challenge and assessment of protection

Mice were immunized with one (Balb/cByJ; Additional file 1A) or two to three (C57BL/6j; Additional file 1B) intravenous (iv) injections of *P. berghei* RAS (16krad, Gammacel 1000 ^137^Cs) at weekly intervals or with one intradermal injection of *P. berghei* CS^5M^ RAS (Additional file 1: Figure S1C). Dose de-escalation of RAS immunization was performed in order to obtain a suboptimal RAS dose to detect possible beneficial effects of CQ (Additional files 1A and 1B). In RAS experiments, RAS-CQ groups received either CQ prophylaxis (chloroquine diphosphate, Sigma-Aldrich) for 10 days (Balb/cByJ –1040 μg base/day oral) or 17 days (C57BL/6j –800 μg base/day intraperitoneal). Efficacy of these prophylactic regimens was established in pilot studies, and they were chosen because of their closest resemblance to the human CPS-protocol. Alternatively, mice were given two subcutaneous injections of 500 μg CQ base, 2 h before and 6 h after each immunization, because this particular regimen was previously shown to improve cross-presentation (Additional file 1B) [21,20].

Furthermore, mice under CQ or MQ prophylaxis were immunized three times at weekly intervals by intravenous administration of 20,000 wild-type *Pb*SPZ (CPS immunization; Additional file 1D). For CPS immunization, CQ (diluted in PBS) and MQ (diluted in DMSO/water for injection) were given orally for 24 consecutive days at dosage 1040 μg base/day (CQ) or 350 μg base/day (mefloquine hydrochloride, Sigma-Aldrich) starting from the first day of *Pb*SPZ administration.

Challenge infections were performed by intravenous injection of 10,000 or 50,000 sporozoites around four or eleven weeks after the end of CQ/MQ prophylaxis. Giemsa-stained blood smears were screened for parasitized red blood cells every other day from days 3–14 and finally on day 21 after challenge. Protection was defined as the absence of blood-stage parasites until day 21 post-challenge (Additional files 1A, B and C).

### *Ex vivo* memory phenotyping and *in vitro* IFNγ responses against sporozoites and blood-stage parasites

Mice were euthanized by isoflurane inhalation after intravenous injection of 50 units heparin. Spleen and liver were collected after perfusion of the liver with 10 ml PBS. Cell suspensions of liver and spleen were made by passage of the organs through a 70-μm nylon cell strainer (BD Labware). Liver cells were re-suspended in 35% Percoll (GE Healthcare) and centrifuged at 800 g for 20 min. Liver and spleen erythrocytes were lysed using 5 min incubation on ice in a lysing solution of ammonium chloride. After erythrocyte lysis, hepatic mononuclear cells (HMC) and splenocytes were re-suspended in RPMI 1640 medium.

Five-colour staining of HMC and splenocytes was performed using monoclonal antibodies purchased from Biolegend: Pacific blue-conjugated anti CD3 (17A2), Peridinin Chlorophyll Protein (PerCP)-conjugated anti CD4 (RM4.5), Alexa fluor 700-conjugated anti CD8a (53–6.7), fluorescein isothiocyanate (FITC)–conjugated anti-CD44, allophycocyanin (APC)– or phycoerythrin-Cy7 (PE-Cy7)-conjugated anti-CD62L (MEL-14). Briefly, 10^6^ cells were re-suspended in cold assay buffer (PBS supplemented with 0.5% bovine serum albumin (Sigma-Aldrich)) and incubated for 30 min at 4°C with the monoclonal antibodies. Cells were fixed with Fix & Perm medium A (Invitrogen) and collected in an assay buffer for measurement.

For the detection of parasite-specific cytokine production, HMC and splenocytes (5x10^5^ cells/well) were co-cultured in complete RPMI 1640 culture medium [29] in the presence of *P. berghei* cryopreserved sporozoites (*Pb*SPZ - 5×10^4^/ml) or infected red blood cells (*Pb*iRBC - 5×10^6^/ml). Exposure to salivary gland preparations from uninfected mosquitoes and uninfected red blood cells (uRBC - 5×10^6^/ml) were used as respective negative controls. Cells were stimulated at 37°C/5%CO2 for 24 hours and Brefeldin A (Sigma-Aldrich) was added during the last four hours (10 μg/ml final concentration). As positive control, PMA (100 ng/ml) and Ionomycin (1.25 μg/ml) (Sigma-Aldrich) were added simultaneously along with Brefeldin A. Cells were harvested after 24-hours *in vitro* stimulation and stained with monoclonal antibodies against CD3, CD4, CD8a and CD44 as indicated above. Fixed and permeabilized cells were stained with PE-conjugated anti-IL-2 (JES6-5H4) and APC-conjugated anti-IFNγ (XMG1.2) in Fix & Perm medium B (Invitrogen) at 4°C for 30 min. Flow cytometry was performed on a 9-color Cyan ADP (Beckman Coulter) and data analysis was performed using FlowJo software (version 9.1; Tree Star), using a gating strategy as described previously [7].

### Quantification of SIINFEKL specific CD8^+^ T cells

Prior to intradermal immunization with 20.000 *P. berghei* CS^5M^ RAS, C57BL/6j mice received 2*10^3^ CD45.1 + OT-1 cells and the RAS-CQ group received a 10-days CQ treatment (1040 μg base/day – oral). Expansion of CD45.1^+^CD8^+^ SIINFEKL cells in liver and spleen was assessed by flow cytometry ten days after immunization as described previously [15] (Additional file 1C).

### Data analysis and statistics

Difference in protection between two groups was tested with a Fisher’s exact test. Overall comparisons between immunized and naïve groups were performed using the Kruskal-Wallis test. Direct comparisons between two groups (RAS versus RAS-CQ or CPS-CQ versus CPS-MQ) were performed by Mann–Whitney *U* test. For the analysis of cytokine production, background responses to salivary glands and uRBC were subtracted from *PbSPZ* and *Pb*iRBC responses, respectively, for each individual mouse. All statistical analyses were performed using PRISM software version 5.0 (Graphpad, San Diego, CA). A p-value of ≤0.05 was considered statistically significant.

## Results

### Effects of chloroquine on RAS immunization

**Figure 1:**
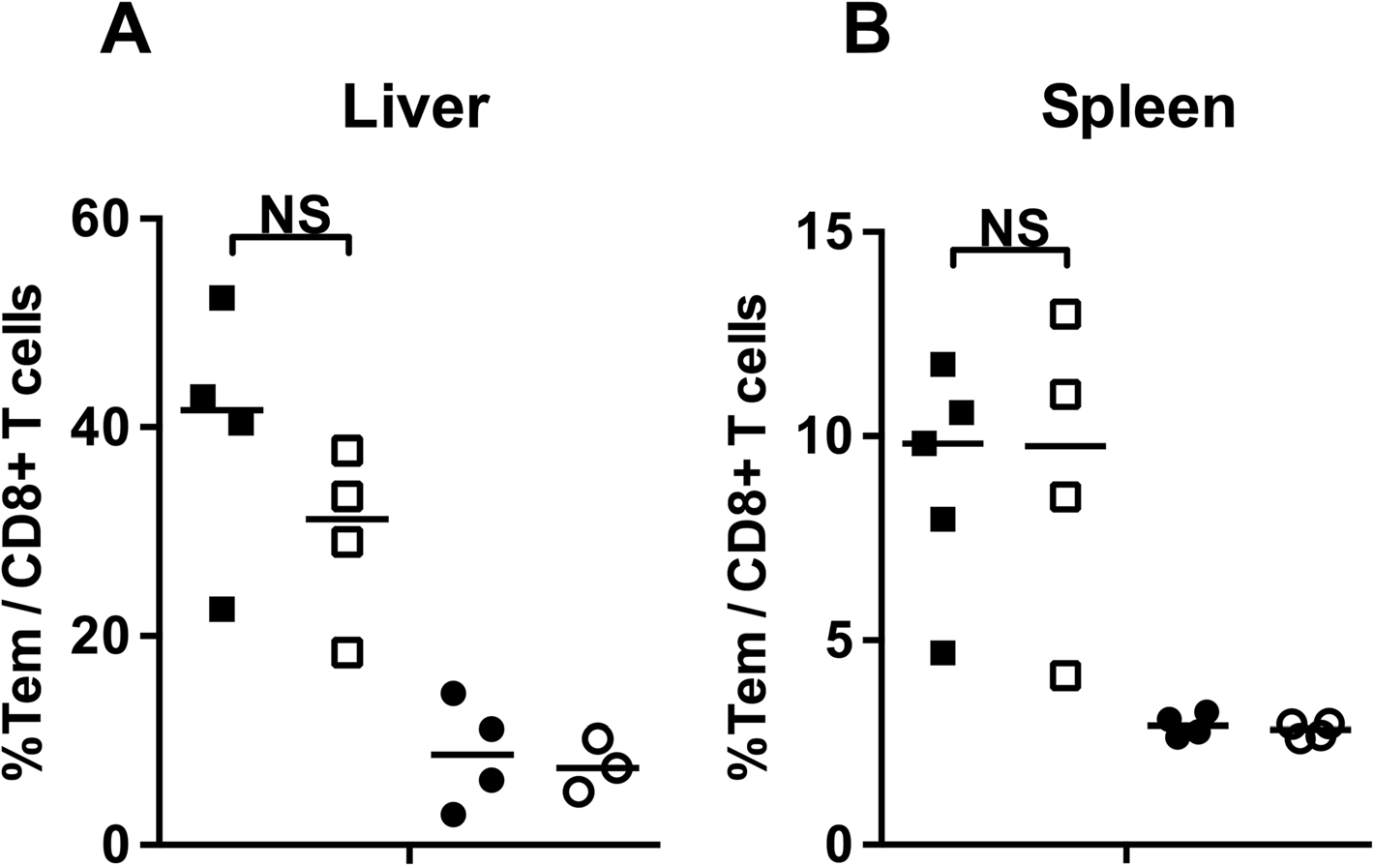
**Frequencies of CD8** ^**+**^ **Tem cells following RAS immunization of C57BL/6j mice under CQ cover.** Percentages of CD8^+^ T cells with effector memory phenotype (CD44^+^CD62L^−^ Tem) were measured one day before challenge by direct *ex vivo* staining in the liver **(A)** and spleen **(B)** of RAS immunized mice (filled squares), RAS immunized and CQ treated mice (open squares), naïve mice (filled circles) and naïve-CQ mice (open circles). Horizontal lines represent group medians. NS = not significant.

First, experiments were performed to investigate whether administration of a prophylactic regimen of CQ improved CD8^+^ memory T cell responses induced by RAS immunization in C57BL/6j mice. *Ex vivo* analysis showed that percentages of CD8^+^ T cells with an effector memory phenotype (CD44^+^CD62L^−^; Tem) in the liver were 4–5 fold higher a day before challenge (C-1) in immunized compared to naïve mice (p = 0.011). However, the percentage of CD8^+^ Tem cells was similar in RAS versus RAS-CQ mice (Figure 1A). Similar patterns were observed in the spleen with three-fold increased Tem levels at C-1 (p = 0.007, Figure 1B).

**Figure 2:**
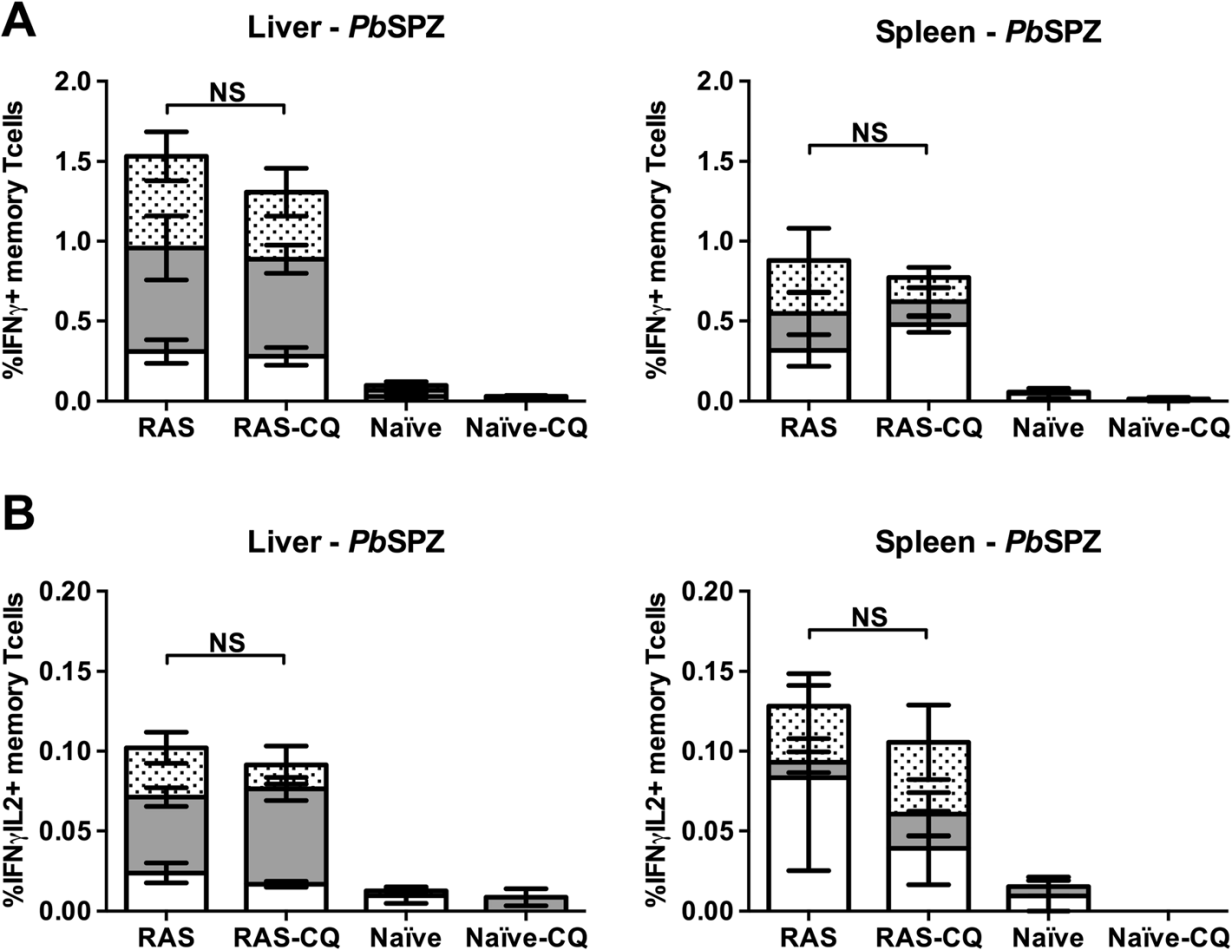
**Sporozoite specific cytokine responses following RAS immunization under CQ cover.** Percentages of IFNγ **(A)** and IFNγ and IL-2 **(B)** producing memory T cells were measured in RAS (n = 5) RAS-CQ (n = 4), naïve control (n = 5) and CQ-control (n = 4) mice, a day before challenge by intracellular staining after *in vitro* re-exposure of liver and spleen cells to *P. berghei* sporozoites (*Pb*SPZ). Responses of CD4^+^ T cells (open area), CD8^+^ T cells (grey area) and CD4^−^CD8^−^ T cells (dotted area) relative to total memory T cell responses are presented. Background responses to salivary glands were subtracted from *Pb*SPZ responses for each individual mouse. Error bars represent standard error of the mean. NS = not significant.

*In vitro* re-exposure of immune cells to *Pb*SPZ showed high levels of IFNγ producing memory T cells in both liver and spleen of RAS-immunized mice (p = 0.003 and 0.027, respectively), which were not increased by additional CQ administration (Figure 2A). Similar observations were made for hepatic and splenic pluripotent memory T cells producing both IFNγ and IL-2, with a major contribution of CD8^+^ cells in the liver (Figure 2B). In summary, additional CQ administration affected neither frequency of RAS-induced CD8^+^ Tem cells, nor sporozoite-specific cytokine production by T cells.

**Figure 3:**
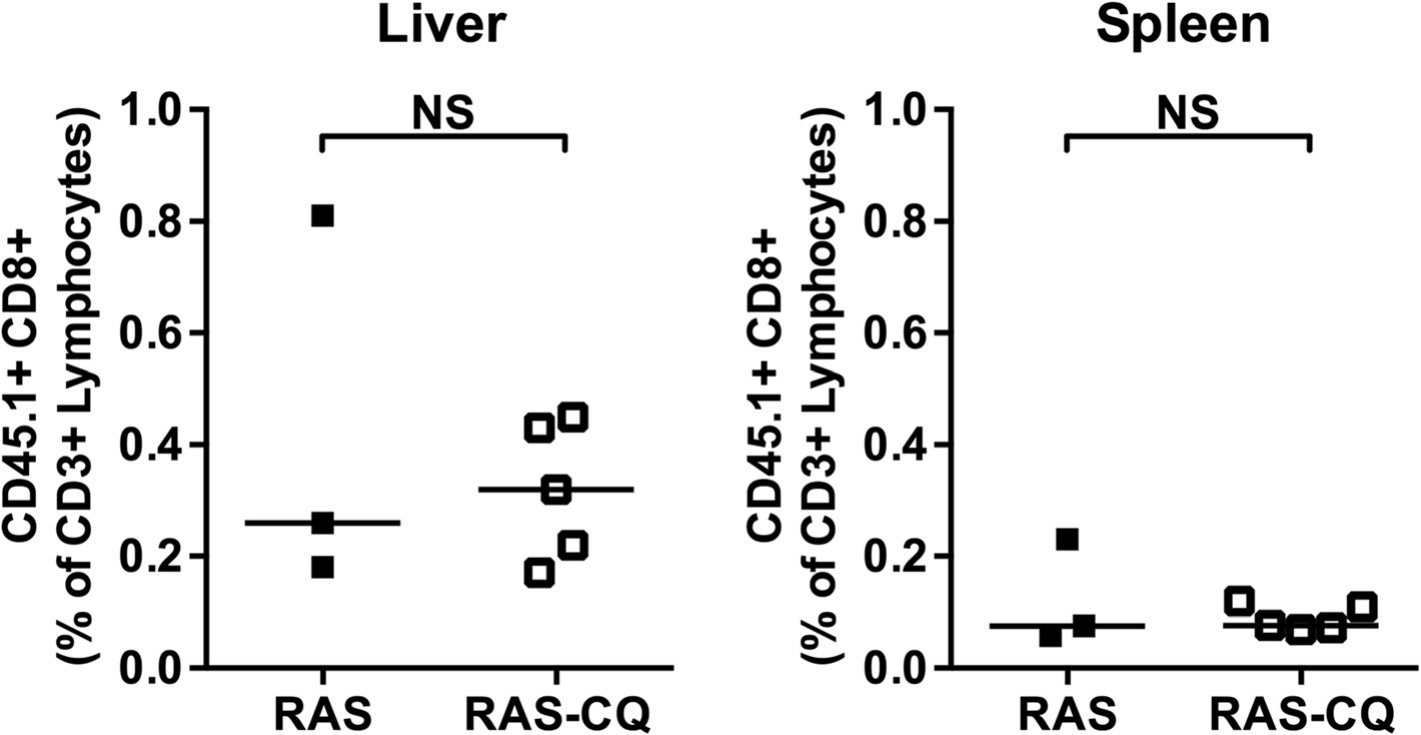
**Frequencies SIINFEKL specific CD8** ^**+**^ **T cells following RAS immunization under CQ cover.** Prior to RAS intradermal immunization with (RAS-CQ) or without chloroquine (RAS), mice received injection of SIINFEKL-specific CD8^+^ T cells. Ten days after a single immunization, expansion of CD45.1^+^CD8^+^ SIINFEKL-specific cells was determined in the liver and spleen of both immunized groups. NS= not significant.

The circumsporozoite (CS) protein is an established target protein of protective immunity in mice and humans [24,30], and the effect of CQ on CD8^+^ T cell responses against a SIINFEKL H-2K^b^ restricted epitope integrated in this protein was studied next. In line with the results above, mice immunized with RAS whilst under CQ cover showed similar percentages of hepatic and splenic CS-specific CD8^+^ T cells compared to untreated mice (Figure 3).

To evaluate a potential effect of CQ on RAS protective efficacy, groups of Balb/cByJ and C57BL/6j mice were immunized with graded numbers of RAS, then challenged and monitored for parasitemia. In both mice strains, reduction of immunization dose resulted in a stepwise decrease in protection that was not influenced by either a prophylactic regimen of daily CQ, or two low doses of CQ at 2 h before and 6 h after each immunization (Table 1).

**Table 1:** **Effect of chloroquine on RAS protective efficacy**

				**No. protected/ no. challenged**	
**Immunization**	**Mouse strain**	**Immunization dose (x10** ^**3**^ **RAS PbSPZ)**	**Inoculations (n)**	**Chloroquine -**	**Chloroquine +** ^**#**^
RAS	Balb/cByJ	1	1	14/16 (88)	13/16 (81)
		0.5	1	4/10 (40)	4/10 (40)
		0.3	1	5/10 (50)	2/10 (20)
	C57BL/6j	10	3	5/5 (100)	4/5 (80)
		4	3	4/5 (80)	4/5 (80)
		1	3	0/5 (0)	1/5 (20)
		4	2	15/23 (65)	14/23 (61)^##^
None	Balb/cByJ	N/A	N/A	1/6 (16)	0/6 (0)
	C57BL/6j	N/A	N/A	0/10 (0)	0/15 (0)

^#^Mice received chloroquine prophylaxis for 10 or 17 days, with the exception of the experiment indicated with ^##^, where mice received two injections of CQ, 2 h before and 6 h after each immunization.

The CQ administered during immunization had no effect on the challenge infection, since all control mice that received CQ-prophylaxis showed the same pre-patent period as untreated naïve mice.

### Comparing chloroquine to mefloquine prophylaxis for CPS immunization

**Figure 4:**
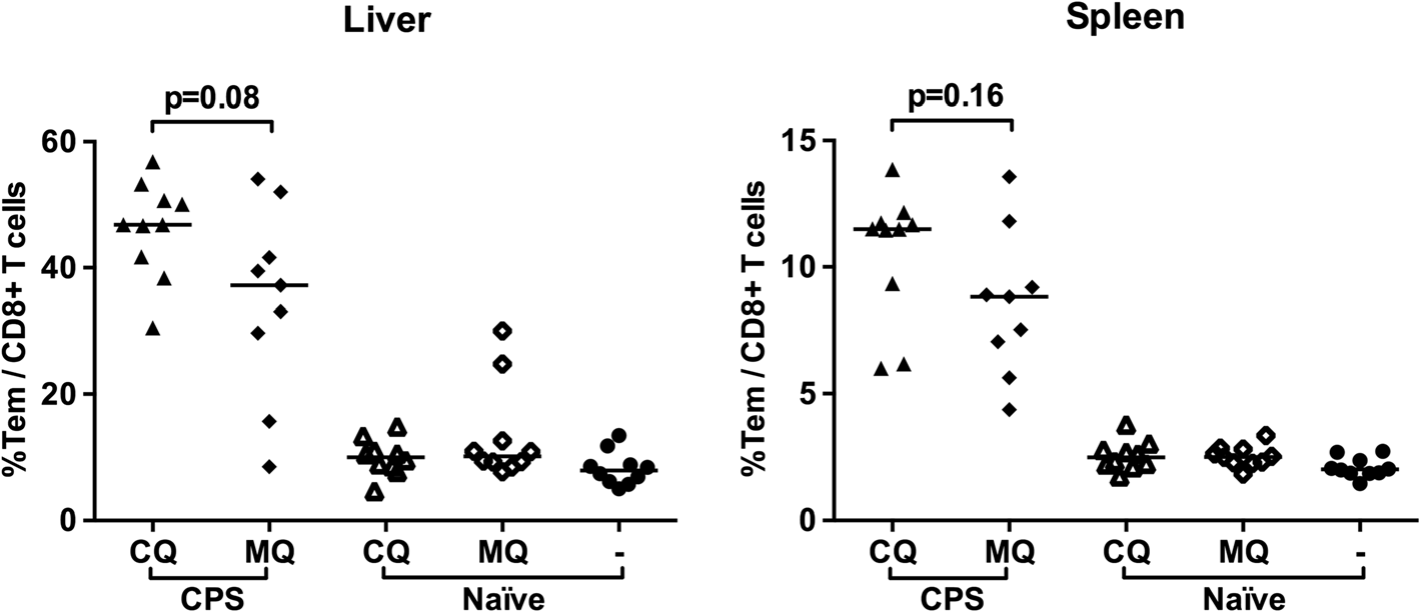
**Frequencies of CD8** ^**+**^ **Tem cells following sporozoite immunization under CQ or MQ cover.** Percentages of CD8^+^ T cells with effector memory phenotype (CD44^+^CD62L^−^ Tem) were measured by direct *ex vivo* staining a day before challenge (C-1) in the liver and spleen of C57BL/6j mice immunized with sporozoites under CQ cover (filled triangle) or MQ cover (filled diamonds), naïve-CQ mice (open triangles), naïve-MQ mice (open diamonds) and untreated naïve mice (filled circles). Horizontal lines represent group medians.

**Figure 5:**
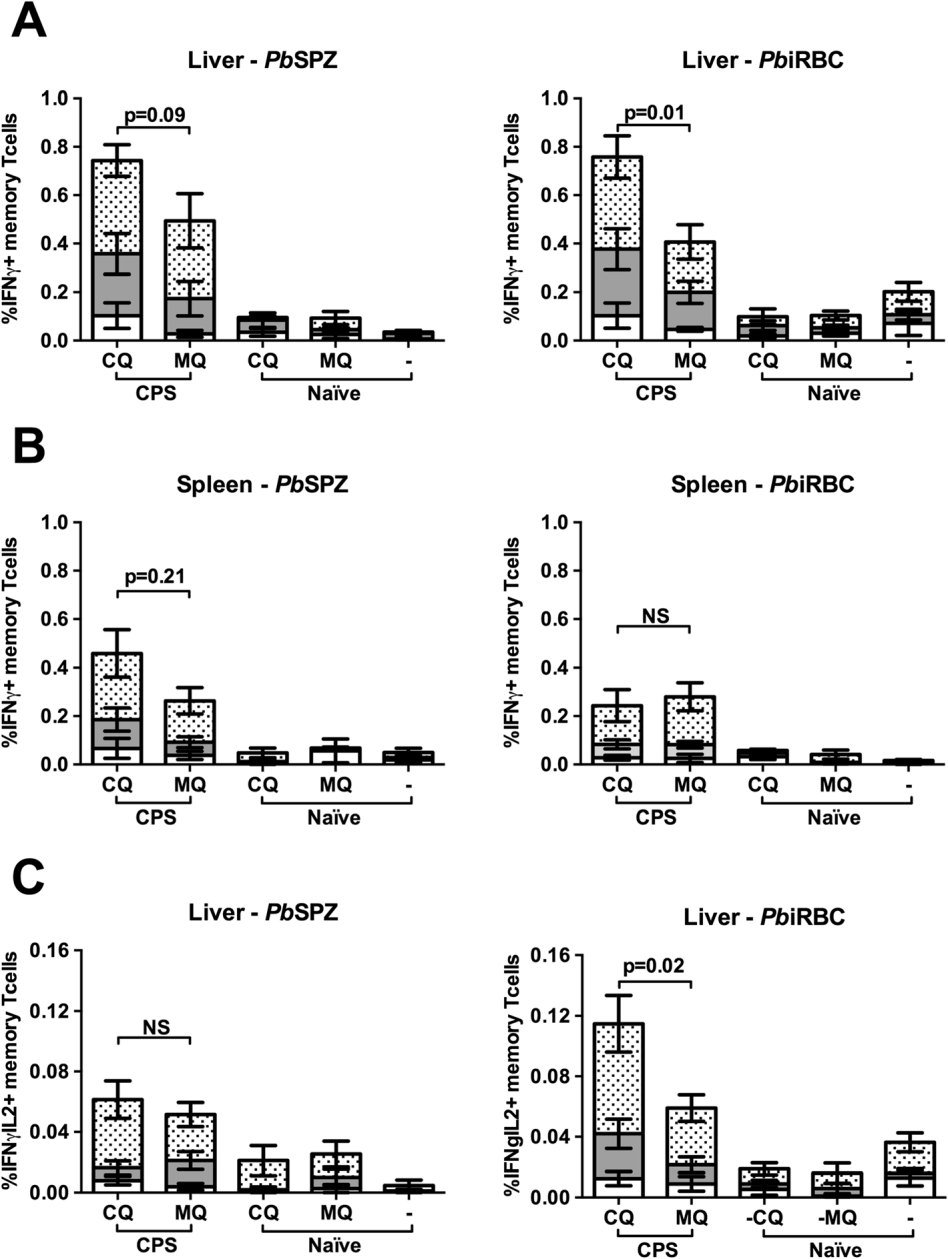
**Sporozoite specific cytokine responses following sporozoite immunization under CQ or MQ cover** ***.*** Percentages of IFNγ producing memory T cells were measured in CPS-CQ (n = 10), CPS-MQ (n-9), naïve-CQ (n = 10), naïve-MQ (n = 10) and no-drug naïve controls (n = 10) a day before challenge by intracellular staining after re-exposure of **(A)** liver and **(B)** spleen cells to *P. berghei* sporozoites (*Pb*SPZ) or infected red blood cells (*Pb*iRBC). Responses of CD4^+^ T cells (open area), CD8^+^ T cells (grey area) and CD4^−^CD8^−^ T cells (dotted area) relative to total memory CD3^+^ T cell responses are presented. **(C)** Hepatic IFNγ and IL-2 responses of memory T cells after re-exposure to *Pb*SPZ and *Pb*iRBC are presented similarly. Background responses to salivary glands and uninfected red blood cells were subtracted from *Pb*SPZ and *Pb*iRBC responses respectively for each individual mouse. Error bars represent standard error of the mean. NS = not significant.

Next, immune responses and protection after CPS-CQ and CPS-MQ immunization were investigated. CD8^+^ Tem levels (Figure 4), IFNγ production upon *in vitro* restimulation with *Pb*SPZ or *Pb*iRBC and pluripotent T cells producing both IFNγ and IL-2 (Figure 5) were significantly increased in immunized compared to control mice.

Although not significant, there was a trend for higher CD8^+^ Tem levels in CPS-CQ compared to CPS-MQ in both liver and spleen (p = 0.08 and 0.16, respectively; Figure 4). T cells isolated from the liver of CPS-CQ mice showed higher IFNγ responses after *in vitro* re-exposure to *Pb*iRBC (p = 0.01) and a trend for higher IFNγ responses to *Pb*SPZ re-exposure (p = 0.09; Figure 5A). In the spleen, a similar trend of higher IFNγ responses in the CPS-CQ group was observed upon re-exposure to *Pb*SPZ but not *Pb*iRBC (Figure 5B). Following *in vitro* re-exposure to *Pb*iRBC, but not *Pb*SPZ, the percentage of hepatic pluripotent memory T cells producing both IFNγ and IL-2 was significantly higher in the CPS-CQ group compared to the CPS-MQ group (p = 0.02, Figure 5C). Thus, CPS-CQ resulted in somewhat higher specific cytokine responses compared to CPS-MQ.

Finally, mice were challenged by intravenous administration of 10,000 sporozoites five or ten weeks after the second booster (day 50 or 100) or with 50,000 sporozoites at day 50. At day 50, 100% of CPS-CQ (13/13) and CPS-MQ (21/21) immunized mice were protected against challenge with low (10 K) or high (50 K) sporozoite dose. All control mice including CQ (10/10) and MQ (10/10) prophylaxis groups developed blood-stage parasitaemia. Postponement of challenge to day 100 resulted in 90% protection (9/10) in both the CPS-CQ and CPS-MQ group (Table 2).

**Table 2:** **Protection by sporozoite immunization under CQ or MQ cover**

	**No. protected/No. challenged (% protection)**		
	**Day 50 (x10** ^**3**^ ***P*** **bSPZ challenge)**		**Day 100 (x10** ^**3**^ ***P*** **bSPZ challenge)**
	**10**	**50**	**10**
CPS-CQ	3/3 (100)	10/10 (100)	9/10 (90)
CPS-MQ	11/11 (100)	10/10 (100)	9/10 (90)
Naïve-CQ	0/5 (0)	0/5 (0)	-
Naïve-MQ	0/5 (0)	0/5 (0)	-
Naïve	0/5 (0)	0/5 (0)	0/5 (0)

Altogether, there was no difference in protection after CPS immunization with either CPS-CQ or CPS-MQ, although cellular responses after whole sporozoite immunization under CQ cover were increased.

## Discussion

Addition of CQ to a *P. berghei* RAS immunization protocol improves neither protection nor parasite-specific CD8^+^ T cells responses. Only slightly reduced T cell responses and similar protective efficacy are found when CPS-CQ is compared to CPS-MQ. This study did not involve an investigation of the effect of CQ on cross-presentation *in vitro*, nor detailed mechanistic antigen presentation studies [31]. Alternatively, more functional readouts were examined such as parasite-specific CD8^+^ T cell responses and protection from challenge infection to assess potential immune enhancing effects of CQ. These combined *P. berghei* data do not provide evidence for significant improvement of whole sporozoite immunization in the presence of CQ and, therefore, indicate that CQ is not responsible for the strikingly higher efficiency of CPS-CQ compared to RAS in humans.

Improved cross-presentation resulting in increased IFNγ production by CD8^+^ T cells has been shown in *in vitro* studies where dendritic cells were pulsed with soluble viral antigen in the presence of CQ [19]. In mice, CQ was shown to enhance cross-presentation of soluble OVA to OT-I cells both *in vitro* and *in vivo* and to improve specific CD8^+^ T cell responses after alum-OVA immunization [21]. An effect of CQ on OVA cross-presentation was observed upon administration of 20 μg, but not 200 μg protein [21], suggesting that immunomodulatory effects of CQ are only beneficial under suboptimal immunization conditions. In the current study, RAS immunization both with and without CQ induces strong cellular responses with similar contribution of CD8^+^ T cells, which translates to equal protection levels. Even after down-titration of RAS immunization dose, which is associated with decreasing protection, clear improvement by CQ remains undetected.

In humans, a single administration of CQ during Hepatitis B booster vaccination significantly improved CD8^+^ T cell response [19]. Despite several reports of enhanced immune responses by CQ in mice and men [21,23,32,19], only one study has reported improved protection; mice immunized with a heat-inactivated influenza virus showed improved survival rates after challenge infection [20]. The significance of improved immune responses for protection thus remains to be further explored.

One cannot assume that improvement of cross-presentation by CQ is applicable to any soluble protein or peptide, or antigens presented in the form of a whole sporozoite. Improvement of cross-presentation of SIINFEKL peptide (OVA_257–264_) and inactivated influenza virus by CQ in mice have been demonstrated [21,20]. Here however, CQ administration during sporozoite immunization with SIINFEKL expressing *P. berghei* sporozoites showed no increase of SIINFEKL-specific CD8^+^ T cells, suggesting that presentation pathways and effects of CQ might differ between pathogens or antigens.

CPS-CQ immunization of C57BL/6j mice requires relatively high and lengthy drug prophylaxis to prevent development of *P. berghei* infection (K. Nganou-Makamdop, unpublished data). As a result, CPS-CQ mice cumulatively receive much more CQ than the two doses of 800 μg chloroquine diphosphate salt that were previously shown to improve cross-presentation [21]. Therefore, the effect of this low CQ-dose regimen on RAS immunization was assessed in a separate experiment, but protection levels were not higher than compared to RAS alone, indicating that the choice of CQ dose was not crucial.

Immune modulating effects of MQ have been reported but do not include cross-presentation [33,25-27]. It cannot be formally excluded that MQ in CPS immunization regimes may have similar properties as CQ. Both CQ and MQ are lysosomotropic agents that limit endosomal acidification [22], which for CQ is known to result in inhibition of lysosomal enzymes that require an acidic pH to function and the fusion of endosomes with lysosomes [34]. As such CQ, but not MQ, has been widely used to study the role of endosomal acidification in cellular processes [35]. CQ has also been studied extensively for its inhibitory effect on autophagy [17], but a recent publication suggests that MQ has similar effects on autophagy [36]. Because both endosomal acidification and autophagy might influence antigen presentation, the effect of MQ on these processes may result in immune modulating effects just as is the case for CQ.

In the absence of evidence for a direct immune-modulating effect of CQ during whole sporozoite immunization, it cannot be ruled out that both CQ and MQ might instead contribute to the efficient induction of protection in an indirect way. A review of rodent sporozoite immunization studies demonstrates the importance of optimal exposure to the entire repertoire of liver stage antigens as occurs during CPS-CQ and CPS-MQ, with reduced protective efficacy if liver stage development is halted by drugs or in the case of RAS or genetically attenuated parasites [37]. Furthermore, some reports show a negative effect of blood-stage parasites on induced pre-erythrocytic CD8^+^ T cell responses by interfering with dendritic cell function [38,1]. By limiting exposure to blood stages during CPS immunization, CQ and MQ might thus have an indirect positive effect on pre-erythrocytic immunity.

## Conclusions

This study does not provide evidence of improved immune responses or protective efficacy by CQ in the *P. berghei* model. Instead, the higher efficiency of CPS compared to RAS in humans might be explained by an indirect effect of CQ through limiting blood-stage exposure after immunization or to an improved breadth of the immune response as a result of increased antigen exposure. In the absence of a clear immune enhancing effect of CQ here, more work is needed to assess whether these findings can be translated to human settings.

## Additional file

Additional file 1:
**Study designs.** The effect of chloroquine (CQ) on immune responses and protection by whole sporozoite immunization was tested in a number of *P. berghei* models. Balb/cByJ (A) and C57BL/6j mice (B) received intravenous (iv) immunizations with radiation attenuated sporozoites (RAS; immunization dose in grey), with or without additional administration of CQ (grey bars). All mice were challenged by iv injection of 10x10^3^ wild type (WT) *P. berghei* sporozoites (spz), and followed up with blood smears for the detection of parasites. (C) C57BL/6j mice were immunized with *P. berghei* CS^5M^ RAS after receiving CD45.1 + OT-1 cells, with or without CQ prophylaxis (grey bar). Expansion of CD45.1^+^CD8^+^ SIINFEKL cells in liver and spleen was assessed by flow cytometry 10 days after immunization. (D) C57BL/6j mice were immunized by iv administration of WT *P. berghei* spz while receiving either CQ or mefloquine (MQ) prophylaxis, then challenged after 50 days with either 10x10^3^ or 50x10^3^ WT *berghei* spz or after 100 days with 10x10^3^ WT *berghei* spz, and followed up with blood smears for the detection of parasites.

## Abbreviations

CQ: Chloroquine
CPS: Chemoprophylaxis and Sporozoites
CS protein: Circumsporozoite protein
HMC: Hepatic mononuclear cell
MQ: Mefloquine
*Pb*iRBC: *P. berghei* infected red blood cells
PBMC: Peripheral blood mononuclear cell
*Pb*SPZ: *P. berghei* sporozoites
RAS: Radiation attenuated sporozoite
Tem: Cells with effector memory phenotype
