# Supplementary figures and images for: Studying the effect of chloroquine on sporozoite-induced protection and immune responses in Plasmodium berghei malaria
Source: Malar J. 2015 Mar 26;14:130. doi: 10.1186/s12936-015-0626-2 (PMC4389414; doi:10.1186/s12936-015-0626-2)

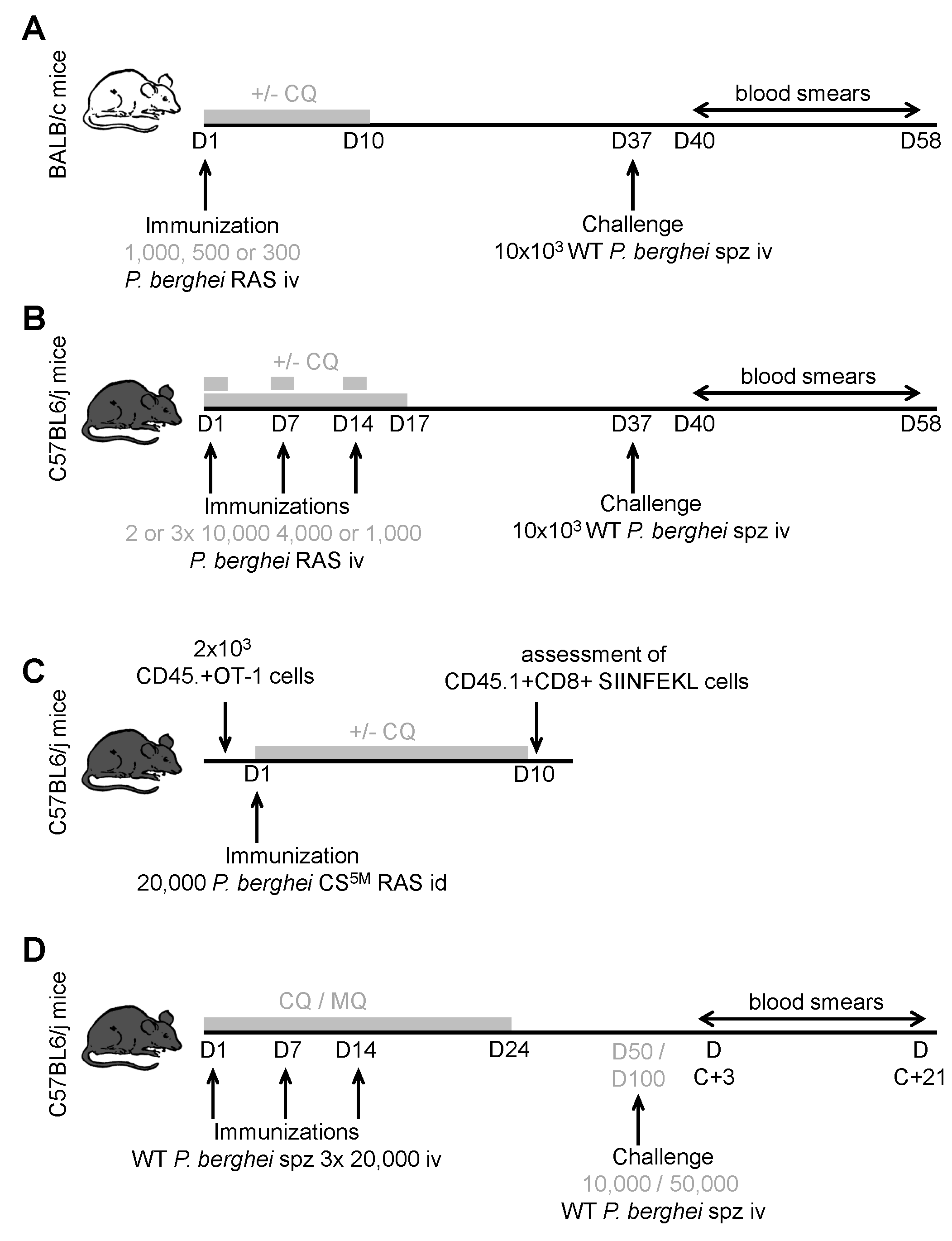

Supplement: Additional file 1: — Study designs. The effect of chloroquine (CQ) on immune responses and protection by whole sporozoite immunization was tested in a number of P. berghei models. Balb/cByJ (A) and C57BL/6j mice (B) received intravenous (iv) immunizations with radiation attenuated sporozoites (RAS; immunization dose in grey), with or without additional administration of CQ (grey bars). All mice were challenged by iv injection of 10x103 wild type (WT) P. berghei sporozoites (spz), and followed up with blood smears for the detection of parasites. (C) C57BL/6j mice were immunized with P. berghei CS5M RAS after receiving CD45.1 + OT-1 cells, with or without CQ prophylaxis (grey bar). Expansion of CD45.1+CD8+ SIINFEKL cells in liver and spleen was assessed by flow cytometry 10 days after immunization. (D) C57BL/6j mice were immunized by iv administration of WT P. berghei spz while receiving either CQ or mefloquine (MQ) prophylaxis, then challenged after 50 days with either 10x103 or 50x103 WT berghei spz or after 100 days with 10x103 WT berghei spz, and followed up with blood smears for the detection of parasites. [file 12936_2015_626_MOESM1_ESM.tif]
